# Novel Cell Permeable Polymers of *N*-Substituted L-2,3-Diaminopropionic Acid (DAPEGs) and Cellular Consequences of Their Interactions with Nucleic Acids

**DOI:** 10.3390/ijms22052571

**Published:** 2021-03-04

**Authors:** Anita Romanowska, Katarzyna Węgrzyn, Katarzyna Bury, Emilia Sikorska, Aleksandra Gnatek, Agnieszka Piwkowska, Igor Konieczny, Adam Lesner, Magdalena Wysocka

**Affiliations:** 1 Faculty of Chemistry, University of Gdansk, Wita Stwosza 63, 80-308 Gdansk, Poland; anita_romanowska@o2.pl (A.R.); emilia.sikorska@ug.edu.pl (E.S.); ognatek@gmail.com (A.G.); apiwkowska@imdik.pan.pl (A.P.); adam.lesner@ug.edu.pl (A.L.); 2 Intercollegiate Faculty of Biotechnology, University of Gdansk Abrahama 58, 80-308 Gdansk, Poland; katarzyna.wegrzyn@biotech.ug.edu.pl (K.W.); katarzyna.bury@biotech.ug.edu.pl (K.B.); igor.konieczny@ug.edu.pl (I.K.); 3 Mossakowski Medical Research Institute, Polish Academy of Sciences, Wita Stwosza 63, 80-308 Gdansk, Poland

**Keywords:** polymers, peptidomimetics, AFM, transfection, molecular modelling

## Abstract

The present study aimed to synthesize novel polycationic polymers composed of *N*-substituted L-2,3-diaminopropionic acid residues (DAPEGs) and investigate their cell permeability, cytotoxicity, and DNA-binding ability. The most efficient cell membrane-penetrating compounds (O2Oc-Dap(GO2)_n_-O2Oc-NH_2_, where n = 4, 6, and 8) showed dsDNA binding with a binding constant in the micromolar range (0.3, 3.4, and 0.19 µM, respectively) and were not cytotoxic to HB2 and MDA-MB-231 cells. Selected compounds used in the transfection of a GFP plasmid showed high transfection efficacy and minimal cytotoxicity. Their interaction with plasmid DNA and the increasing length of the main chain of tested compounds strongly influenced the organization and shape of the flower-like nanostructures formed, which were unique for 5/6-FAM-O2Oc-[Dap(GO2)]_8_-O2Oc-NH_2_ and typical for large proteins.

## 1. Introduction

Gene transfection can be defined as the transmission of DNA to regulate or induce specific gene expression in the target cells or organs. This mechanism is very important in biosciences, pharmaceutics, and clinical applications. In the past decades, several transfection systems including viral and non-viral vectors have been developed. DNA chains are negatively charged polymers, repelling each other owing to the intrachain and interchain electrostatic repulsion among fragments, a major drawback of which is low transfection efficiency [1,2,3,4]. To enhance the effective transfer of a plasmid or short linear molecule of nucleic acid through the cell membrane, two major barriers need to be overcome. The first problem is the negative charge of DNA molecules, which should be neutralized or masked. The second issue, in some cases, is the size of DNA molecules, especially large vectors or plasmids. There are several reports describing the methodology to cause DNA condensation, which results in the concurrent reduction in size and charge. This strategy mimics the natural processes of genome organization by positively charged proteins or small cationic compounds such as bivalent or multivalent metal cations and polyamines [5,6].

In the late 1980s, a group of positively charged molecules referred to as cell-penetrating peptides (CPPs) was developed by many research groups [7,8]. Owing to their high content of basic amino acid residues (Arg and Lys) and positive charge at physiological pH, CPPs passively diffuse across the lipid bilayer, chiefly owing to endocytosis. Moreover, CPPs can cross the plasma membrane at low micromolar concentrations in vivo and in vitro without using any receptors and without causing any significant membrane damage [9,10]. Another advantage of using CPPs for the therapeutic delivery of numerous molecules, including DNA, is the lack of toxicity in comparison with other cytoplasmic delivery systems, such as liposomes and polymers [11]. Additionally, the strong positive charge of most CPPs neutralizes the DNA, resulting in its condensation, which is beneficial for cellular delivery [12,13].

**Figure 1:**
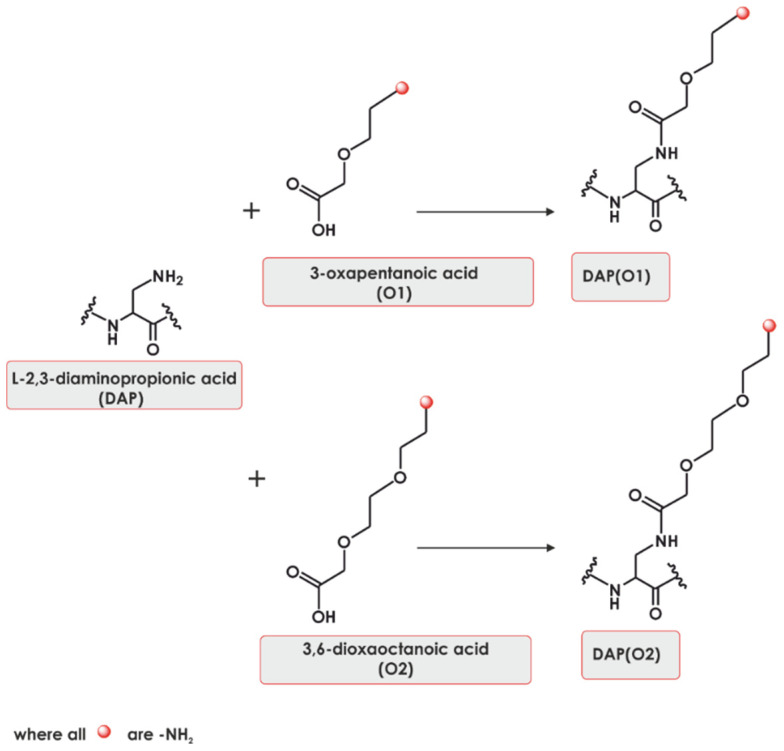
Examples of *N*-substituted L-2,3-diaminopropionic acid residues (DAPEGs).

Recently, our group reported the synthesis of a novel class of peptidomimetics [14]. Such molecules are synthesized using two building blocks; the beta amino group of diaminopropionic acid was decorated by a functionalized oxa acid (Figure 1). We were able to manipulate the length and properties of the side chain and functional groups, producing novel amino acid mimetics. The deconvolution of a 400-member library composed of *N*-substituted L-2,3-diaminopropionic acid residue (DAPEG) building blocks facilitated the selection of an efficient and selective fluorogenic probe or substrate of neutrophil serine protease **4**. The obtained substrate is cleaved by neutrophil serine protease **4** in an efficient and selective manner [14]. More recently, a fluorogenic probe for a trypsin-like subunit of 20S proteasome, composed of DAPEG building blocks, was synthesized and found to be useful in the diagnostics of bladder cancer [15].

**Figure 2:**
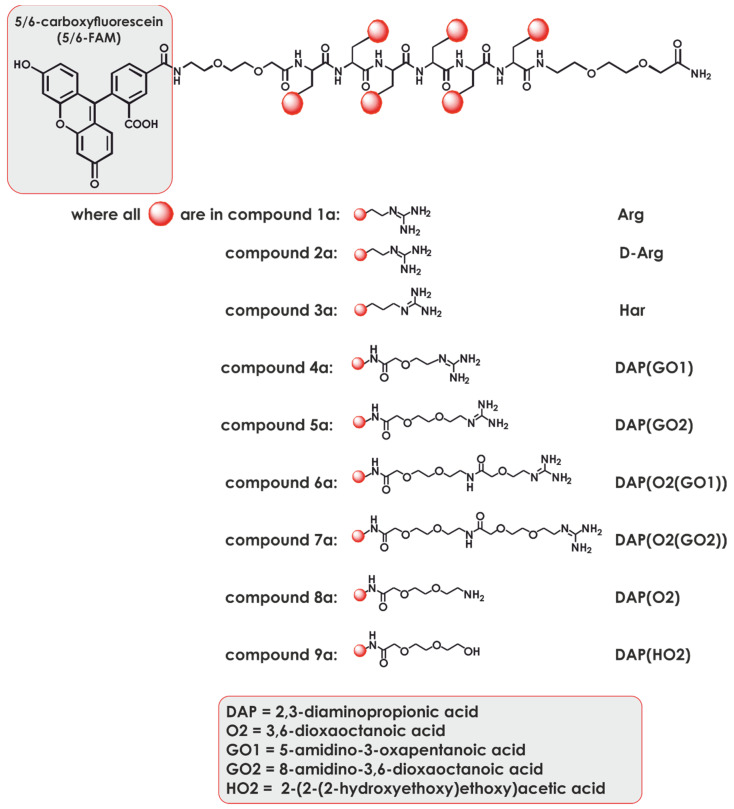
Chemical formulae of compounds from series **1**.

In the present study, we synthesized homopolymers containing a different number of L-2,3-diaminopropionic acid residues (**2**–**8**) that were decorated by functionalized oxa acids (see Figure 2) with a variety of side chain groups. Moreover, three compounds containing arginine (Arg) and its analogs (d-arg and homoarginine (Har)) in their structure were synthesized and treated as controls. Thus, nine different molecules were obtained; their N-terminal amino groups were labeled with a 5/6-carboxyfluorescein succinimidyl ester (5/6-FAM) fluorophore, and the molecules were selected using 5/6-carboxytetramethylrhodamine succinimidyl ester (5/6-TAMRA) derivatives. The aim of this study was to synthesise the panel of DNA binding polymers that, in a complex with target DNA, are able to efficiently penetrate the cell membrane allowing gene deliery. To do so, cytotoxicicty studies and DNA binding efficacy, along with cell penetration assay were performed. Based on a combination of the above, the selected compounds were subjected to transfection experiments using a model GFP vector, and the most effective was selected for further structural studies.

## 2. Results and Discussion

### 2.1. Synthesis

In total, nine compounds were synthesized using a synthetic method (see Figure S1) [14]. All synthesized compounds were labeled with a 5/6-FAM fluorophore and two with an N-terminal 5/6-TAMRA group. The physicochemical characteristics of all compounds are listed in Table 1 and Table 2.

### 2.2. Cell Permeability and Localization

**Figure 3:**
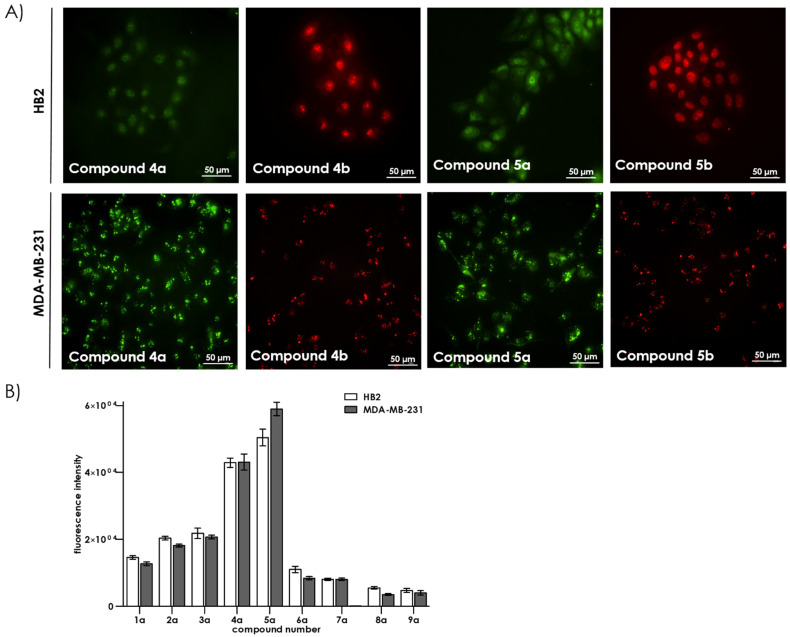
(**A**) Cell membrane permeability in HB2 and MDA-MB-231 cells incubated with 10 µM of compounds **4a**, **4b**, **5a**, and **5b** for 24 h. Magnification 20×. (**B**) Fluorescence intensity in HB2 and MDA-MB-231 cells incubated with 10 µM of compounds **1a**–**9a**.

**Figure 4:**
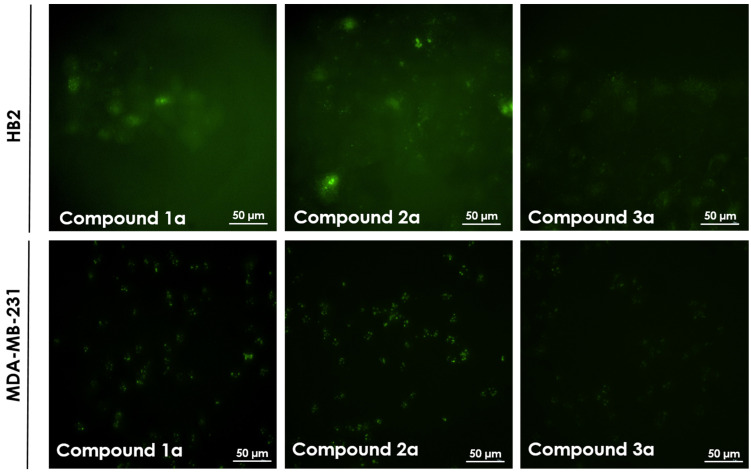
Cell membrane permeability in HB2 and MDA-MB-231 cells incubated with 10 µM of compounds **1a**, **2a**, and **3a** for 24 h. Magnification 20×.

Preliminary cell permeability experiments facilitated the identification of three groups of compounds. The first group was visible in the nuclei of HB2 or MDA-MB-231 cells incubated with the compounds at micromolar level (10 µM) (Figure 3). The first group includes compound **4** and its derivatives (**4a** and **4b**) and the family of compound **5** (**5a** and **5b**). Compounds **4** and **5** are similar in structure and are composed of six residues of diaminopropionic acid (Dap) decorated with side chains of different lengths, terminating with the same highly positive guanidine moiety. However, compounds **5a** and **5b** showed a significantly higher intensity of nuclear penetration/accumulation than compounds **4a** and **4b**. Both compounds display unique cellular localization. The second group of compounds (**1a**, **2a**, and **3a**) are able to cross the cell membrane, and their presence is partially visible in the cytoplasm in the form of granules (see Figure 4). The compounds in the third group (**6a**, **7a**, **8a** and **9a**) were not visible by fluorescence microscopy indicating that they did not cross the cell membrane. We believe that such effective nuclear localisation of the first group of tested compounds is due to the effective mimicking of the nuclear localisation sequence (NLS). Such sequence consists of several positively charged lysines or arginines present in the protein structure. Additionally, side chain length of the polymer molecules seems to be a crucial factor that is optimal for compounds belonging to first group of polymers.

Notably, most of these compounds did not show cytotoxicity at the concentration used in the cell experiments (10 µM). However, compounds **1a**, **2a**, and **3a** were slightly cytotoxic; the number of cells incubated with these compounds was slightly more reduced than those in control (Figure S2).

**Figure 5:**
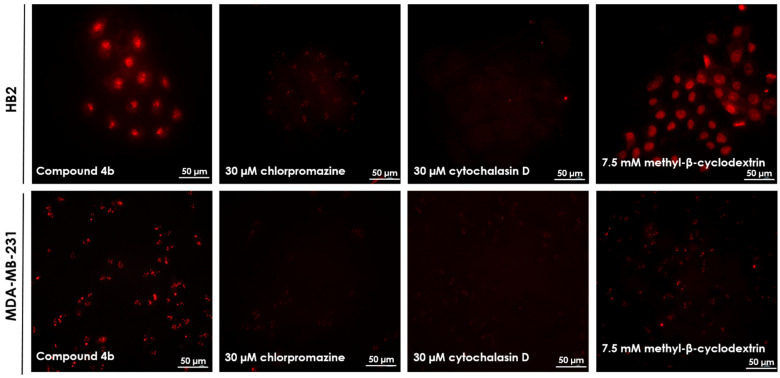
Cellular uptake study of compound **4b** in HB2 and MDA-MB-231 cells (incubation time 24 h, 37 °C). All other details are provided in individual images. Magnification 20×.

Next, we aimed to explore the effect of active processes (such as endocytosis) and passive, energy-independent mechanisms (such as pore formation) on the cellular internalization of compounds **4b** and **5b**. Incubation of HB2 or MDA-MB-231 cells cultured with compounds **4b** and **5b** at 4 °C, at which all energy-dependent uptake processes are significantly reduced, resulted in an almost complete inhibition of **4b** and **5b** uptake into the cells. These findings indicate that the cellular internalization of the peptide largely occurs through an active, energy-dependent uptake mechanism. To examine whether this energy-dependent uptake process includes a specific endocytic route, the cells were preincubated with a set of endocytosis inhibitors (methyl-β-cyclodextrin, cytochalasin D, and chlorpromazine) [16,17,18]. As seen in Figure 5, cytochalasin D and chlorpromazine significantly reduced the fluorescence intensity of compound **4b** within the cells; however, methyl-β-cyclodextrin did not reduce itsfluorescence intensity, indicating that it did not affect the uptake process. These findings suggest the presence of a mixed mechanism of uptake of compound **4b**. For compound **5b** (data not shown), the same observation was made, indidating that both compounds follow the clathrin-dependent and actin-dependent endocytosis.

Nucleic acid binding ability of compounds **1**–**9** by electrophoretic mobility shift assay (EMSA), surface plasmon resonance (SPR), and microscale thermophoresis (MST).

**Figure 6:**
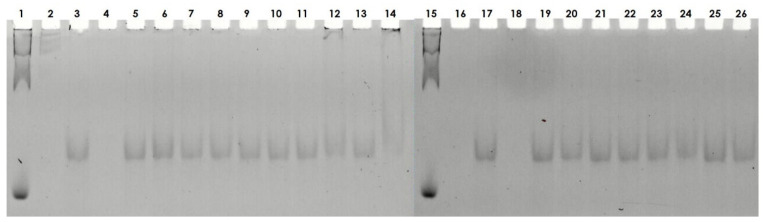
DNA-binding activity of compounds **1**–**9** was evaluated using a polyacrylamide electrophoretic gel mobility shift assay. (lanes **1**, **15**) DNA marker; (lanes **3**, **17**) dsDNA 76 bp; (lanes **5**, **6**) compound **1** in two different N/P ratios—0.2:1 and 1.5:1 (charge peptidomimetic/charge dsDNA), respectively; (lanes **7**, **8**) compound **2** in various N/P ratios—0.2:1 and 1.5:1, respectively; (lanes **9**, **10**) compound **3** in various N/P ratios—0.2:1 and 1.5:1, respectively; (lanes **11**, **12**) compound **4** in various N/P ratios—0.2:1 and 1.5, respectively; (lanes **13**, **14**) compound **5** in various N/P ratios—0.2:1 and 1.5:1, respectively; (lanes **19**, **20**) compound **6** in various N/P ratios—0.2:1 and 1.5:1, respectively; (lanes **21**, **22**) compound **7** in various N/P ratios—0.2:1 and 1.5, respectively (charge peptidomimetic/charge dsDNA); (lanes **23**, **24**) compound **8** in various N/P ratios—0.2:1 and 1.5:1, respectively; (lanes **25**, **26**) compound **9** in various N/P ratios—0.2:1 and 1.5:1, respectively; (lanes **2**, **4**, **16**, **18**) intentionally empty lanes. N/P is defined as ratio of positively chargeable polymer amine (N = nitrogen) groups to negatively charged nucleic acid phosphate (P) groups.

**Figure 7:**
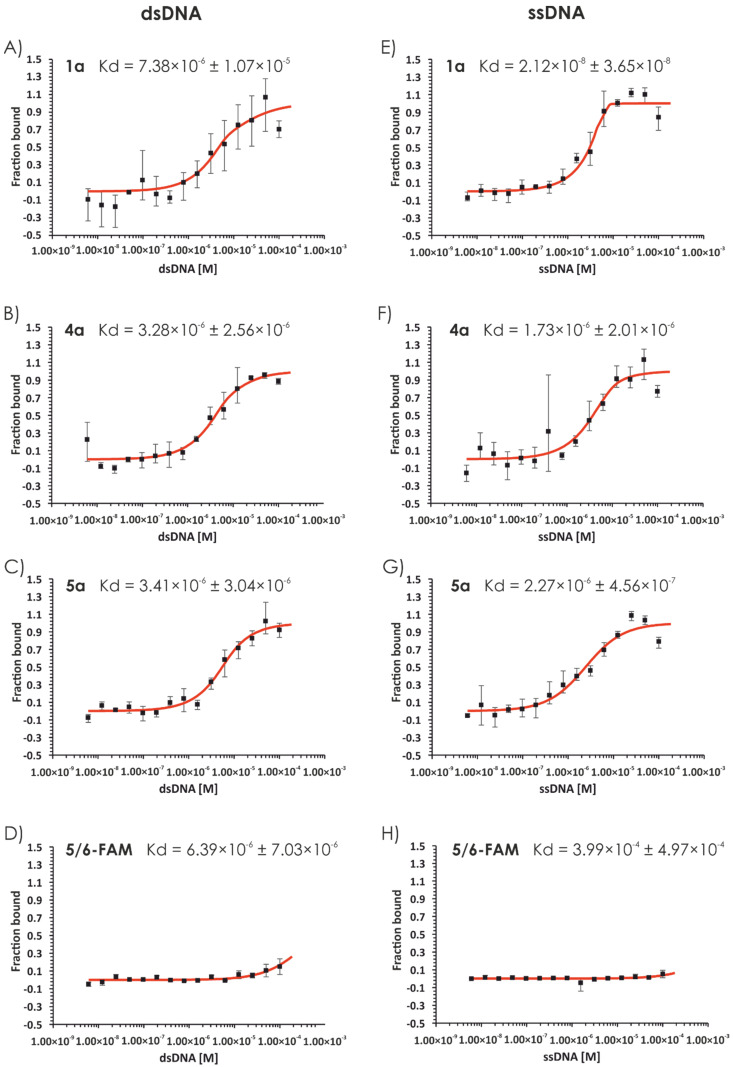
Microscale thermophoresis (MST) analysis of dsDNA binding by 5/6-FAM-labeled compounds. The MST analysis. FAM-labeled compounds **1a** (**A**), **4a** (**B**), **5a** (**C**), and ssDNA binding by 5/6-FAM-labeled compounds **1a** (**E**), **4a** (**F**), **5a** (**G**) was performed using the Monolith NT.115 instrument (NanoTemper). Binding was measured between increasing concentrations (6.1 nM–200 µM) of dsDNA (76 bp fragment) and 0.437 µM of the indicated compounds labeled with 5/6-FAM. Control experiments were performed with DNA and 5/6-FAM dye (**D**,**H**).

Owing to the nuclear localization of the compounds **4a**, **4b**, **5a**, and **5b** and their highly positive charge, we decided to investigate the nucleic acid-binding ability of compounds **1**–**9**. Initially, we used a short linear model 76 bp dsDNA fragment containing the sequence of beta-actin (*Homo sapiens*). Polyacrylamide gel separation of the compounds incubated with the model DNA resulted in significant retardation, indicating the interaction of the synthesized compounds (**1**–**9**) with dsDNA (Figure 6). This is visible for compounds **4** (lines 13, 14) and **5** (lines 15, 16) and slightly visible for compounds **6** (21, 22), **7** (23, 24), and **8** (25, 26). The minimal C/P ratio resulting in forming complex of each compound is provided in Table S1. Preliminary surface plasmon resonance (SPR) analysis performed using two different concentrations (50 and 100 µM) of the compounds with dsDNA as a ligand indicated that at the lower concentration, compound **5** showed the highest DNA-binding ability, followed by compound **4** and compounds **1**–**3**, which are guanidine-rich polymers (Figure S3). At the concentration of 100 µM, compound **4** showed stronger DNA binding, and it is followed by compounds **1**–**3**. Substitution of guanidine groups by amino groups (compound **8**) significantly reduced the strength of interaction. Moreover, the analog of compound **5** with six hydroxyl moieties on its side chain (compound **9**) did not show any DNA binding (Figure S4). For compounds with high DNA-binding ability, the binding constant was measured using the SPR technique (Table 1). Both compounds **4** and **5** displayed similar binding constants (see Table 1) in the micromolar range (8.8 ± 2.1 µM for compound **4** and 1.8 ± 0.9 µM for compound **5**). To confirm these findings in an alternative system, we performed similar experiments using microscale thermophoresis (MST); the results are presented in Table 1. The binding constant values were in the micromolar range and increased in the following order: **4a** < **5a** < **1a** (Figure 7).

**Figure 8:**
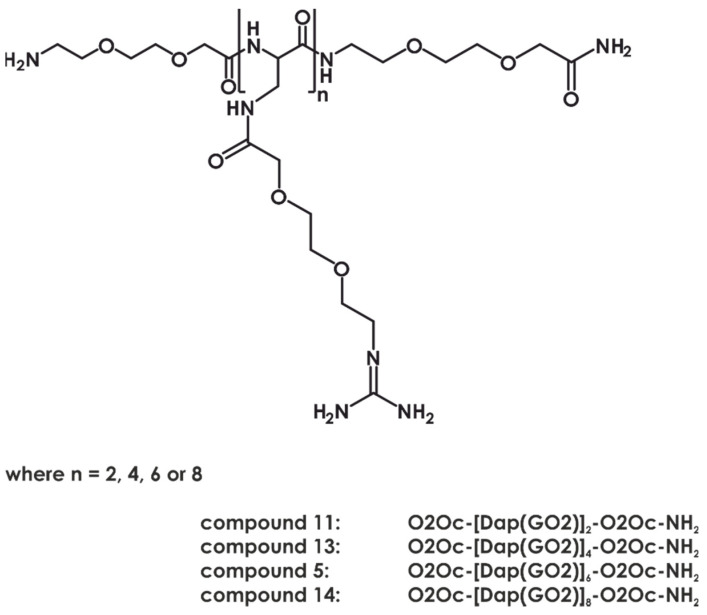
Chemical formulae of second generation compounds with varying lengths of the main chain.

**Figure 9:**
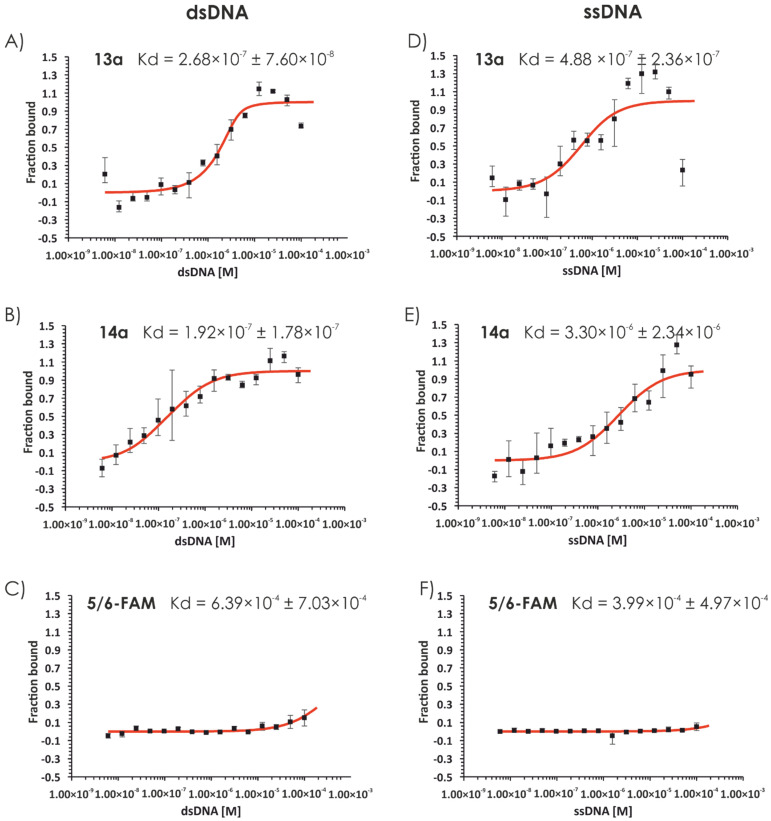
Microscale thermophoresis (MST) analysis of dsDNA binding by 5/6-FAM-labeled compounds. The MST analysis of dsDNA binding by 5/6-FAM-labeled compounds **13a** (**A**) and **14a** (**B**), and ssDNA binding by 5/6-FAM-labeled compounds **13a** (**D**) and **14a** (**E**) was performed using the Monolith NT.115 instrument (NanoTemper). Binding was measured between increasing concentration (6.1 nM–200 µM) of dsDNA (76 bp fragment) and 0.437 µM of the indicated compounds labeled with 5/6-FAM. A control experiment was performed with DNA and 5/6-FAM dye (**C**,**F**).

Considering two factors: cell permeability (in which the leading compound was **5a**) and DNA binding (in which compound **5a** was one of the most potent nucleic acid binders), we decided to synthesize a second set of the analogs with varying numbers of monomers in its structure (Figure 8). Thus, compounds **11**, **13**, and **14** (two, four, and eight residues of Dap(GO2), respectively) and their fluorescent derivatives (**11a**, **13a**, **14a**, and **14b**) were obtained. The cell permeability assay indicated that hexamers (**5a**) and octamers (**14a** and **14b**) efficiently penetrate the membrane and localized in the nucleus. Moreover, the DNA binding constants of the tested compounds according to the MST assay were in the micromolar and submicromolar range (Figure 9). Tested compounds displayed no or low cytotoxicity (compound **14**; Figure S5). The cytostatic effect was evaluated using 5-ethynyl-2′-deoxyuridine incorporation assay.

### 2.3. Transfection

**Figure 10:**
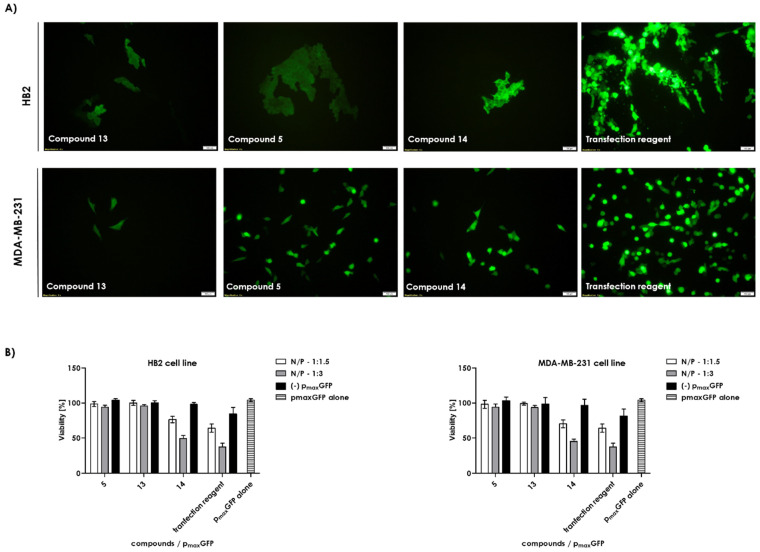
(**A**) Fluorescence imaging of HB-2 and MDA-MB-231 cells transfected with pmaxGFP plasmid mixed with compounds **5**, **13**, **14**, or the commercially available transfection reagent (control). (**B**) Cytotoxicity of plasmid pmaxGFP:compound complex in HB2 and MDA-MB-231 cells at the concentration of the complex equal to 4.62 × 10^−10^ M.

Next, we used the novel compounds from the second generation series (**5**, **13**, **14**) as transfection agents (Figure 10A). The model plasmid encoding green fluorescent protein (PmaxGFP), size 3486 bzp) was used for transfection. Most cells in the system with these compounds and with the control (a commercially available transfection reagent the commercially available transfection reagent ViaFect) showed green fluorescence that indicates sucesfull transfection. The highest fluorescence intensity was observed for compound **5**, followed by that for compounds **14** and **13**. The post-transfection survival rate was comparable for compounds **13** and **5**, whereas compound **14** showed 30% cytotoxicity. The control reagent showed the highest cytotoxicity; 50% of cells transfected with the control did not survive, but the fluorescent intensity of the system was comparable with that observed with compound **5** at its optimal concentration (Figure 10B).

**Figure 11:**
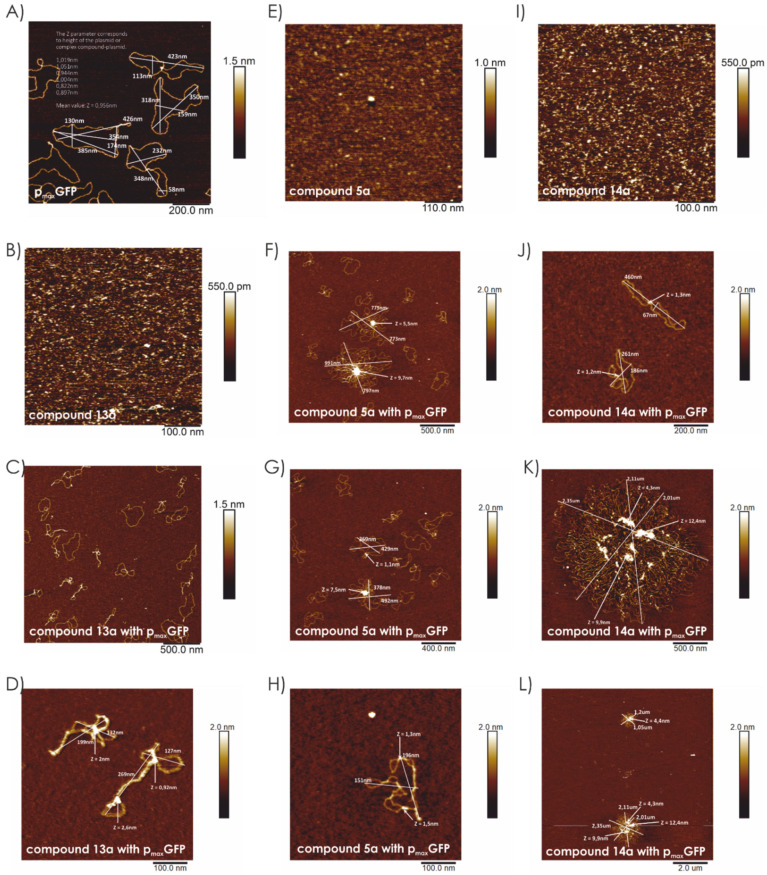
Atomic force microscopy (AFM) findings. (**A**) plasmid p_max_GFP; (**B**) compound **13a**; (**C**) compound **13a** with plasmid p_max_GFP–N/P 0.2:1, 2.5 µm; (**D**) compound **13a** with plasmid p_max_GFP–N/P 0.2:1, 500 nm; (**E**) compound **5a**; (**F**) compound **5a** with plasmid p_max_GFP–N/P 0.2:1, 2.5 µm; (**G**) compound **5a** with plasmid p_max_GFP–N/P 0.2:1, 2.5 µm; (**H**) compound **5a** with plasmid p_max_GFP–N/P 0.2:1, 500 nm; (**I**) compound **14a**; (**J**) compound **14a** with plasmid p_max_GFP–N/P 0.2:1, 1 µm; (**K**) compound **14a** with plasmid p_max_GFP–N/P 0.2:1, 2.5 µm; (**L**) compound **14a** with plasmid p_max_GFP–N/P 0.2:1, 10 µm.

In the above experiment, we expected a strong correlation between the strength of interaction with DNA and efficient membrane translocation of the compounds tested. However, the relatively low transfection efficacy of compound **13** did not correlate with its high DNA binding constant and rapid cellular internalization. This discrepancy may be explained by the structure and size of the DNA-compound complex. Positively charged compounds interact with DNA by forming electrostatic interactions with the phosphate groups of the DNA backbone. We analyzed the shape and organization of such complexes in the form used for transfection using atomic force microscopy (AFM). As seen in Figure 11B,E,I, the compound itself is observed as a small dot despite its number of building blocks, and plasmid p_max_GFP is observed to form several conformations (Figure 11A). Figure 11C,D show compound **13a** complexed with DNA in an N/P ratio of 0.2:1 (charge peptidomimetic/charge p_max_GFP); the structure formed seems to be typical for small cationic molecules such as polyamines and metal ions (e.g., Ca^2+^ and Mg^2+^) or poliArg [19,20]. Compound **13a** binds to the DNA sequence causing plasmid condensation, leading to a size reduction.

Compound **5a** complexed with DNA, creating flower-like structures [21], with an average size reaching 900 nm (Figure 11F–H). A limited number of single-plasmid compound **5a** structures are also observed in the investigated system. The analysis of the system in which compound **14a** was complexed with the plasmid showed a shift towards larger multiplasmid particles (referred to as connected coils) [22], with sizes up to 2350 nm (Figure 11J–L). However, single-plasmid complexes are also present in the system.

Compound **14a** forms large compact structures or aggregates with a defined high-density core, where several compound **14a** molecules are bound to several plasmid molecules; the structure resembles bacterial chromosome organization, with its average size reaching 2.35 µm. These structures are observed with a charge ratio of 0.2:1, in which the charge of DNA dominates over the charge of compound **14a**. Such a structure seems to act as a DNA scavenger able to bind all DNA molecules present in the system tested.

In general, the observed flower-like structures are highly looped with multiple crossover points. The complexity and compactness of the structure tends to increase with increasing number of monomers in the analyzed compounds (e.g., see compounds **13** versus **14**).

**Figure 12:**
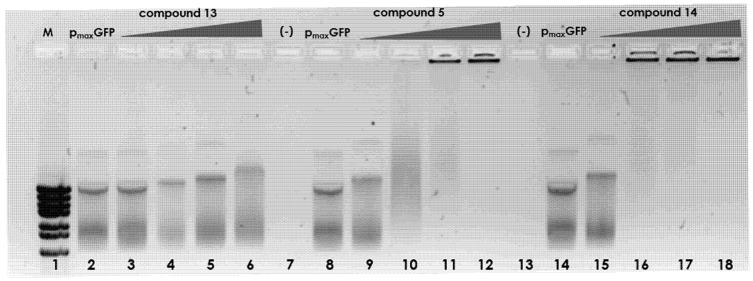
DNA-binding activity of compounds **13**, **5**, and **14** was evaluated using an electrophoretic gel mobility shift assay. (lane **1**) DNA marker; (lanes **2**, **8**, **14**) pmaxGFP plasmid; (lanes **3**–**6**) compound **13** in various N/P ratios—0.2:1, 1:1, 1.5:1, and 3:1 (charge peptidomimetic/charge plasmid), respectively; (lanes **9**–**12**) compound **5** in various N/P ratios—0.2:1, 1:1, 1.5:1, and 3:1, respectively; (lanes **15**–**18**) compound **14** in various N/P ratios—0.2:1, 1:1, 1.5:1, 3:1; (lanes **7**, **13**) intentionally empty lanes.

The above findings were confirmed by agarose gel electrophoretic separation of the formed complexes. As seen in Figure 12, the titration of the plasmid using increasing concentrations of compound **13** (lines 3–6) results in relatively minor changes in the electrophoretic mobility of the complexes formed. However, the findings for plasmid complexes with compound **5** were significantly different; the charge ratio of 1.5:1 and greater resulted in the formation of large complexes that were unable to penetrate the agarose gel. When the charge ratio reached the highest value (3:1), the complex was unable to penetrate the gel and remained in the well. This may be explained by either overall charge reduction and/or mass increase in the formed complex. This finding is consistent with the AFM images indicating that compound **13** causes intramolecular condensation of the plasmid, whereas compounds **14** and **5** are able to link together several molecules of the plasmid. Such large complexes were formed at a lower concentration of compound **14** (above charge ratio 1), which confirms the presence of the large structures observed in the AFM images. This is the possible reason for the low transfection efficacy mediated by compound **14**.

To further understand the role of the peptidomimetics in DNA condensation, theoretical models representing interactions of compounds **13** and **14** with DNA were created. Due to the complexity of such calculations, we decided to analyze only for two distinct systems and exclude compound **5** that behaved in a mixed mode. The peptidomimetics selected for molecular dynamics (MD) simulations were composed of the same amino acid-like units but differed from each other in sequence length, which is expected to affect the way the dsDNA is condensed.

**Figure 13:**
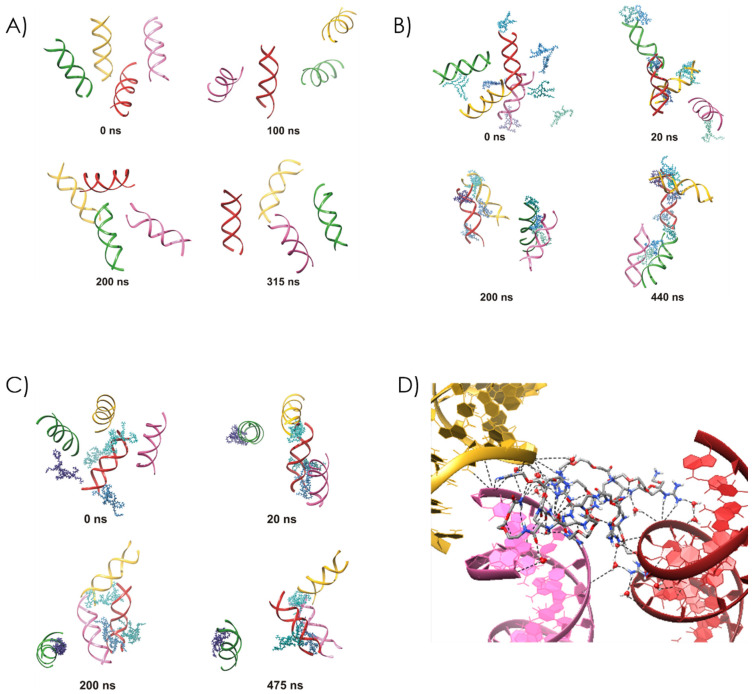
Snapshots from molecular dynamics (MD) simulations examining DNA condensation in a system (**A**) without a peptidomimetic, (**B**) in the presence of compound **13**, and (**C**) in the presence of compound **14**. The four DNA helices are colored gold, green, pink, and red. The peptidomimetic molecules are colored in various shades of blue. (**D**) Interactions between a single molecule of compound **14** and three DNA double helices at the final step of the simulation.

The results showed that both peptidomimetics induced DNA condensation, and the process began in the early steps of molecular dynamics MD simulations. The peptidomimetics–DNA complexes were stabilized by a hydrogen bond network, including water-bridged hydrogen bonds and salt bridges. In contrast, the control simulation with no peptidomimetics showed no DNA aggregation (Figure 13).

Molecular dynamic simulation of polymer–DNA interaction.

**Figure 14:**
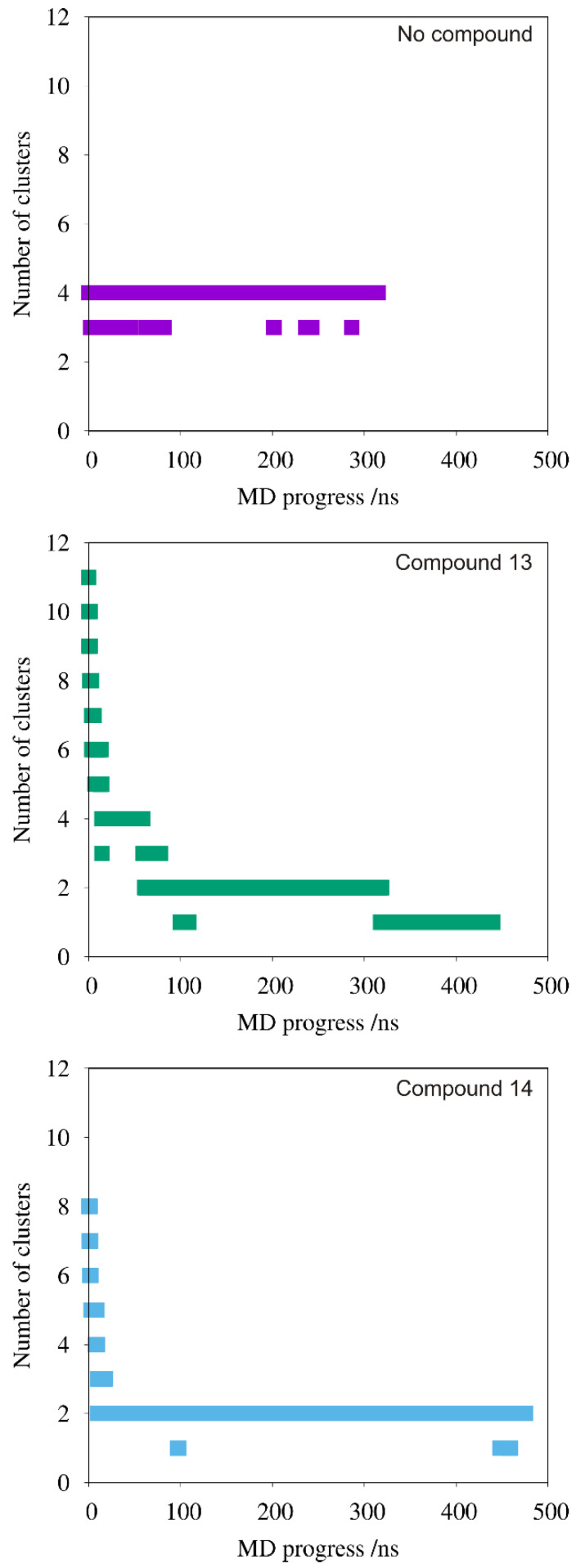
Progression of DNA condensation shown as aggregate numbers versus molecular dynamic (MD) progress. The peptidomimetic and dsDNA molecules at a distance ≤3.5 Å were considered to be in a cluster. The number of clusters at the beginning of a simulation depends on the initial number of DNA and peptide molecules in the system.

Both polymer-containing systems had a 0.2:1 compound: DNA charge ratio, indicating that in the MD simulation, twice as many compound molecules are needed to achieve the same condition for compound **13** as for compound **14**. This leads to a greater dispersion of the positive charges in the simulation system and affects the subsequent steps of DNA condensation. With compound **13**, the DNA condensation occurs in a clear stepwise manner. In the beginning, the formation of the dsDNA-compound complexes is frequently observed. This complexation shields the charges on the DNA phosphate groups and diminishes the inter-strand phosphate–phosphate repulsion, which facilitates binding of other dsDNA units. In detail, the first 20 ns of the simulation lead to a peptide-mediated condensation of two DNA double helices, whereas two others remain unaggregated until ~80 ns of the MD simulation. For the next 250 ns, two compound-bridged dsDNA assemblies are already observed. Finally, after about 330 ns of the MD simulation, all dsDNA molecules condense together to form a stable four-DNA bundle. In contrast, the longer side chain of compound **14** favors faster DNA condensation. The extended arms of compound **14** can quickly capture neighboring DNA helices during the initial steps of the MD simulation, simultaneously initiating dsDNA compound binding and dsDNA condensation. As seen in Figure 13C, three molecules of compound **14** mediate the aggregation of three dsDNA helices within the first 20 ns of the MD simulation, whereas the fourth one with one the associated peptide molecule stays away in an unaggregated form. This configuration remains unchanged for the rest of the trajectory. The fourth DNA double helix forms occasional contacts with the three-DNA bundle, but does not bind to it permanently, which is well reflected in the number of clusters forming within the whole trajectory (Figure 14). Remarkably, the length of compound **14** enables interactions of a single peptide molecule with as many as three DNA helices, which is not observed for its shorter counterpart (Figure 13D).

The findings of the above experiments suggest that despite the superior properties of compound **14**, it forms complexes with a size exceeding that required for effective cell penetration. The compound **13** complexes are relatively small, and cell penetration of the compound alone is low. Thus, the highest transfection efficacy observed for compound **5** is expected because the size of the DNA; compound **5** complex seems to be optimal for cell membrane crossing. To the best of our knowledge, the large complexes recorded for compound **14** have been reported until now only for large nuclear proteins with dedicated functions, such as viral proteins [22] and bacterial proteins [23]. Such large and dense DNA aggregates may resemble or even mimic a simplified neutrophil extracellular traps (NETs) network [24] or organization of bacterial DNA [25].

Synthesized compounds prove to be good DNA binders comparable to hexaArg with binding constants in the micromolar range. An exceptional feature of such compounds is their ability to rapidly translocation into the nucleus of the cells tested, which is not reported for reference compounds. Additionally, polymer length-dependent DNA structures are unique for such types of molecules. Finally, the polymer transfection efficacy is strongly influenced by length of the molecule used, and it is optimal for hexamer molecules.

The obtained new molecules being polymers of *N*-substituted L-2,3-diaminopropionic acid belong to the class of cell-penetrating inert polymers. Due to DNA binding properties, few of them facilitate gene delivery to the cells studied. In our opinion, the utilization of the above compounds is not limited to non-cytotoxic transfection, but they could also be employed as DNA binding agents. In a broader perspective, they could be DNA sensors or scavengers.

## 3. Materials and Methods

### 3.1. Chemistry

#### Compound Synthesis

All compounds were synthesized on the amide TentaGel S RAM resin (Rapp Polymer, Tubingen, Germany) by Fmoc solid-phase methods. Compounds **1**, **2**, and **3** were synthesized using an automated microwave peptide synthesizer (Liberty Blue, CEM, Matthews, NC, USA); the following reagents were used: Fmoc-O2Oc-OH (where O2Oc is 8-amino-3,6-dioxaoctanoic acid) and Fmoc-l-Arg(Pbf)-OH, or Fmoc-d-arg(Pbf)-OH, or Fmoc-Har(Pbf)-OH. Compounds **1a**, **2a**, and **3a** were synthesized by coupling 5/6-FAM to peptidomimetics **1**, **2**, and **3**, respectively, as described below. To obtain compounds **4**, **5**, **6**, **7**, **8**, **9**, **12**, **13**, and **14**, initially, Fmoc-O2Oc-OH and Fmoc-l-Dap(Mtt)-OH (where l-Dap-OH is l-2,3-diaminopropionic acid) were used for the main chain synthesis (Fmoc-O2Oc-[Dap(Mtt)]_n_-O2Oc-R) on an automated microwave peptide synthesizer (Liberty Blue). The scale of each synthesis was 0.1 mmol; amino acid derivatives were dissolved in 0.2 M *N*,*N*-dimethylformamide (DMF). Piperidine (20%) in DMF was used for deprotection; 0.5 M *N*,*N*′-diisopropylcarbodiimide in DMF was used as activator; 1.0 M OXYMA in DMF was used as activator base; and DMF was used as main wash. A protocol without final Fmoc deprotection was used. After automatic microwave synthesis, 4-methyltrityl (Mtt) protection groups were removed using the procedure described previously [26] (1% trifluoroacetic acid (TFA) in dichloromethane (DCM) with addition of 2% 1,2-ethanedithiol [EDT]). The mixture was added to compounds **4**, **5**, **6**, **7**, **8**, **9**, **12**, **13**, and **14** and stirred for 15 min. The resin was washed with DCM between each wash. This procedure was repeated until no increase in absorbance at 410 nm was noted. Then, 1% *N*,*N*-diisopropylethylamine (DIPEA) in DMF solution was added to each portion of the peptidyl resin three times for 10 min. Next, GO2 (compounds **5**, **12**, **13**, **14**), GO1 (compound **4**), O2 (compound **8**), HO2 (compound **9**), and Mtt-O2Oc-NH_2_ (compounds **6** and **7**) were coupled to each -NH_2_ moiety of Fmoc-O2Oc-(Dap)_n_-O2Oc-R using equimolar amounts of Amino acid/TBTU/OXYMA/DIPEA in DMF/DCM/2-*N*-methyl-2-pyrrolidone (NMP; 1:1:1, *v*/*v*/*v*) solution. Completeness of the coupling was controlled using Kaiser and chloranil tests. After adding the Mtt-O2Oc moiety to compounds **6** and **7**, Mtt protection groups were removed, and GO1 and GO2 were coupled to resulting free amino groups of compound **6** and **7**, respectively. After confirming the coupling, Fmoc deprotection from *N*-terminal peptidomimetics were performed by using 2 g piperidine in 96 mL NMP with the addition of 2 g 1,8-Diazabicyclo [5.4.0] undec-7-ene for 15 min; during deprotection, the peptidyl resin was washed with DMF, and the whole procedure was repeated six times. Next, fluorophores such as 5/6-FAM or 5/6-TAMRA were attached to the *N*-terminal amino group; a mixture of DIPEA in molar excess of the fluorophore (1:3) in DMF was used. After completing the synthesis, the peptidomimetics were cleaved from the resin, using a TFA/phenol/H_2_O/thioanisole/EDT mixture (82.5:5:5:5:2.5, *v*/*m*/*v*/*v*/*v*). The purity of the synthesized compounds and the accuracy of synthesis were confirmed using ultra-performance liquid chromatography using the Nexera X2 LC-30AD system (Schimadzu, Tokyo Japan) equipped with a Phenomenex column (150 × 2.1 mm), with a grain size of 1.7 µm (peptide XB-C18) equipped with a UV-Vis detector and a fluorescence detector. A linear gradient from 2% to 80% B within 15 min was applied (A: 0.1% TFA; B: 80% acetonitrile in A). The peptidomimetics were monitored at 216 nm. The molecular weights of the synthesized compounds were confirmed by analysis of the mass spectra which were recorded on a Biflex III MALDI-TOF mass spectrometer (Bruker Daltonics, Bremen, Germany) using 2,5-dihydroxybenzoic acid as a matrix.

### 3.2. Biology

#### 3.2.1. Cell Culture

Healthy cell line HB2 (human breast epithelial cells) was obtained from Merck (Hamburg, Germany), and cancer cell line MDA-MB-231 (human breast cancer epithelial cells) was obtained from ATCC (Lomianki, Poland). The MDA-MB-231 and HB2 cells were cultured at 37 °C in 5% CO_2_ in Dulbecco’s Modified Eagle Medium (DMEM; high glucose) supplemented with 10% fetal bovine serum (FBS) and 1% penicillin–streptomycin solution containing 100 units of penicillin and 100 µg/mL of streptomycin. The HB2 cell line requires the addition of 5 µg/mL insulin and 5 µg/mL alcoholic hydrocortisone solution.

#### 3.2.2. Fluorescence Microscopy

HB2 and MDA-MB-231 cells were seeded on 24-well plates at a density 1.5 × 10^4^/well and 4 × 10^4^/well, respectively, and incubated in 0.5 mL complete medium for 48 h. Subsequently, cells were washed with phosphate-buffered saline (PBS), and fresh medium with a fluorescently labeled peptide was added to each well at a concentration of 10 µM. For nucleus staining a DAPI solution (ThermoFisher Scientific, Waltham, MA, USA, country R37606) in PBS was employed in conditions recommended by the supplier. After the incubation (2 h or 24 h), cells were washed thoroughly with PBS, and then a phenol red-free culture medium (FluoroBrite, DMEM) was added. Subsequently, the cells were examined using an Olympus IX51 fluorescence microscope (Olympus, Tokyo, Japan) using appropriate filters: blue for DAPI, green for FITC, and red TAMRA. Next, the images were merged and colocalization of the dyes’ emission were analyzed.

#### 3.2.3. Cytotoxicity Assay: MTT

Cell viability against cell-penetrating compounds was detected by the MTT assay. The HB2 and MDA-MB-231 cells were seeded into 96-well plates at a density of 5 × 10^3^/well and 7 × 10^3^/well, respectively, and incubated in complete medium (100 µL/well) for 48 h. Then, the medium was replaced with fresh medium (100 µL/well), and solutions of test compounds at various concentrations (1, 10, and 50 µM) were added; the cells were incubated for 24 h. Cells incubated in media without any test compounds were used as control. After incubation, medium containing test compounds was removed, and 225 µL fresh medium with 25 µL 3-(4,5-dimethylthiazol-2-yl)-2,5-diphenyltetrazolium bromide (MTT) was added (0.5 mg/mL per well). After incubation at 37 °C for 4 h, supernatants were removed, and the formazan crystals were dissolved overnight with dimethyl sulfoxide (DMSO; 150 µL/well). Results were analyzed using a microplate reader (SPECTROstar Nano, BMG LABTECH, Ortenberg, Germany) at 570 nm and 690 nm.

#### 3.2.4. Cytotoxicity Assay–CCK-8

Cell viability against cell-penetrating peptidomimetics was detected using the cell counting kit-8 (CCK-8) assay (Sigma Aldrich, Poznan, Poland). The HB2 and MDA-MB-231 cells were seeded into 96-well plates at a density of 5 × 10^3^/well and 7 × 10^3^/well, respectively, and incubated in complete medium (100 µL/well) for 48 h. Then, the medium was replaced with fresh medium (100 µL/well), and solutions of compounds at various concentrations (1, 5, 10, 20, 50, and 100 µM) were added; the cells were incubated for 2 h or 24 h. Cells incubated in media without any additives were used as control. After incubation, 10 µL/well of 2-(2-methoxy-4-nitrophenyl)-3-(4-nitrophenyl)-5-(2,4-disulfophenyl)-2H-tetrazolium, monosodium salt (WST-8) reagent was added according to the manufacturers’ instructions, and the mixture was incubated at 37 °C for 4 h. Cell viability was analyzed on the basis of formazan absorbance using a microplate reader (SPECTROstar Nano, BMG LABTECH, Ortenberg, Germany) at 450 nm.

#### 3.2.5. Cytotoxicity of Peptidomimetic–Plasmid p_max_GFP Complexes

In this experiment, second-generation compounds (compounds **5**, **13**, and **14**) were used as transfection reagents. See transfection procedure in methodology below. After gene expression, the medium was removed and cells were lysed with 0.5 M NaOH (100 µL/well), and the fluorescence intensity was analyzed using a microplate reader at excitation and emission wavelengths of 488 and 510 nm, respectively.

#### 3.2.6. Endocytosis Inhibitors

HB2 and MDA-MB-231 cells were seeded into 24-well plates at a density of 1.5 × 10^4^/well and 4 × 10^4^/well and incubated overnight at 37 °C. The medium was then replaced with FBS-free medium and cultured for 24 h more. Afterwards, the cells were treated with endocytosis inhibitors cytochalasin D (final concentration: 1, 2, 5, 10, 20, and 30 µM), chlorpromazine (final concentration: 1, 2, 5, 10, 20, and 30 µM), and methyl-β-cyclodextrin (final concentration: 1, 2.5, 5, and 7.5 mM) for 30 min before adding 10 µM a compound **4b** or **5b**. The cells were incubated with the compounds for 2 h and later washed three times with PBS; the cells were observed under an Olympus IX51 fluorescence microscope (Olympus, Tokyo, Japan). After observation, cells were lysed with 0.5 M NaOH (500 µL/well), and the fluorescence intensity was analyzed using a microplate reader at excitation and emission wavelengths of 558 and 575 nm, respectively.

#### 3.2.7. Transfection

In this experiment, second-generation (GO2)_n_ compounds (compounds **5**, **13**, and **14**) were used as transfection reagents. HB2 and MDA-MB-231 were seeded in 96-well plates at a density of 5 × 10^3^/well and 7 × 10^3^/well in complete medium and grown until 60–70% confluency. Afterwards, a transfection complex solution was prepared: 200 ng of p_max_GFP plasmid from Lonza (Basel, Switzerland) was mixed with (GO2)_n_ peptidomimetics in various N/P ratios (1.5:1 and 3:1; charge peptidomimetic/charge plasmid) (where N/P is defined as the ratio of positively chargeable polymer amine (N = nitrogen) groups to negatively charged nucleic acid phosphate (P) groups) and CaCl_2_ (4 mM per well)) and incubated for 30 min at room temperature until complex formation. The DNA concentration was determined using UV readout at 260/280 nm. The concentration of polymers using UV signal was at 216 nm. As control, ViaFect (Promega, Walldorf, Germany), a commercially available transfection reagent, was used according to the manufacturers’ instructions. Then, the medium in the plate was replaced with serum- and antibiotic-free medium (90 µL/well), and the transfection complex solution was added to each well (10 µL/well). After 5 h incubation, the medium was replaced with 100 µL/well of fresh medium supplemented with 10% FBS, and the plate was incubated for another 48 h to allow for gene expression. Results were observed by fluorescence microscopy.

#### 3.2.8. Electrophoretic Mobility Shift Assay

- DNA polyacrylamide gel electrophoresis. The DNA-binding activity of compounds **1a**–**9a** was examined using electrophoretic mobility shift assay. The dsDNA; (76 bp) model fragment) was mixed with the peptidomimetics in various N/P ratios (0.2:1 and 1.5:1; charge peptidomimetic/charge dsDNA); after incubating for 30 min, 4 μL of loading buffer was added to the samples. The DNA–peptidomimetic complexes were resolved by 8% polyacrylamide gel electrophoresis, and the migrated DNA was visualized under UV light using the fluorescent dye Midori Green.
- Agarose gel electrophoresis. To test the interactions of compounds **5**, **13**, and **14** with DNA, electrophoretic mobility shift assay was performed. The p_max_GFP plasmid was mixed with the peptidomimetics in various N/P ratios (0.2:1, 1:1, 1.5:1, and 3:1; charge peptidomimetic/charge plasmid); after incubating for 30 min, 4 μL of loading buffer was added to the samples. The plasmid–peptidomimetic complexes were resolved by 0.7% agarose gel electrophoresis, and the migrated DNA was visualized under UV light using the fluorescent dye Midori Green.

#### 3.2.9. Molecular Dynamics

##### All-Atom Self-Assembly Simulations

The interaction of selected compounds (compounds **13** and **14**) with DNA was studied by performing all-atom MD simulations using the GPU/CUDA-accelerated implementation of PMEMD in AMBER 16 [27]. Non-standard residues were modelled with the XLEAP module. The point charges were optimized by fitting them to the ab initio molecular electrostatic potential (6–31G* basis set, GAMESS 2013-ab initio molecular electronic structure program) [28] for two different conformations, followed by consecutive averaging of the charges over all conformations, as recommended by the RESP protocol [29]. The initial system for simulations consisted of four double helical DNA molecules with a sequence 5′-ATTGGCAATGAGCGGTTCCG-3′, modeled in an ideal B-form, without and with added selected compounds. In systems with the peptides, a peptidomimetic: DNA charge ratio of 1:5 was maintained to replicate the experimental conditions. Therefore, four molecules of compound **14** and eight molecules of compound **13** were added to the simulation boxes containing four double helical DNA 20-mers in a random position. Each system was solvated and neutralized by adding sodium and chloride ions. The concentration of free salt ions was approximately 100 mM. The 315–475 ns simulations at 300 K with isotropic pressure coupling and 2 fs time step were conducted under periodic boundary conditions with long-range electrostatic interactions evaluated by the particle Mesh Ewald (PME) summation, and a cut-off of 10 Å was used for van der Waals interactions. The SHAKE algorithm was used to constrain bonds involving hydrogen. The temperature was maintained using the Langevin coupling scheme with a friction coefficient of 1 ps^−1^, whereas a Berendsen barostat maintained the reference pressure set to 1.0 bar. The analyses were performed with the CPPTRAJ module of AmberTool v16 (San Francisco, CA, USA). The aggregation process was investigated with the GROMACS 2019.4 suite (Groningen, The Netherlands) [30]. The visualizations were created using UCSF Chimera v1.15 (San Francisco, CA, USA) [31].

#### 3.2.10. Surface Plasmon Resonance Analysis

Standard surface plasmon resonance (SPR) analyses using a Biacore T200 (GE Healthcare, Warsaw, Poland) were performed essentially as described in the manufacturers’ manual. DNA binding by all tested compounds was studied using a 5′-biotinylated 76 nt ssDNA or 76 bp dsDNA fragment containing the sequence of β-actin (*Homo sapiens*), immobilized on a streptavidin matrix-coated sensor chip SA (GE Healthcare, Warsaw, Poland). All oligonucleotides were commercially synthesized (oligo.pl, Poland; Figures S3 and S4). The dsDNA was immobilized on the sensor surface to yield a final value of ~50 RU for dsDNA or ~100 RU. Experiments were performed at 25 °C, and the running buffer was HBS-EP (150 mM NaCl, 10 mM HEPES (pH = 7.4), 3 mM EDTA, and 0.05% Surfactant P20). In binding experiments, the buffer flow rate was set to 15 µL/min, and in kinetic experiments, the buffer flow rate was 30 µL/min. The data were analyzed using Biacore T200 evaluation software (GE Healthcare, Warsaw, Poland). The results are presented as sensorgrams obtained after subtracting the background response signal from a reference flow cell and from a control experiment with buffer injection.

#### 3.2.11. Microscale Thermophoresis

Microscale thermophoresis was performed using the Monolith NT.115 instrument (NanoTemper Technologies GmbH, Munich, Germany). Binding between dsDNA (76 bp) or ssDNA (76 nt) fragments and test compounds labeled with 5/6-FAM was measured. A control experiment was performed with DNA and 5/6-FAM dye. A 16-step dilution series of DNA (400 µM) was prepared in EDBS buffer (25 mM Tris-HCl (pH = 8), 4% (*w*/*v*) sucrose, 4 mM DTT, and 80 μg/mL BSA). Next, 10 µL of 5/6-FAM-labeled compounds diluted in EDBS buffer were added to 10 μL of DNA solution (1:1 dilution series) to reach a final concentration of 0.437 µM. The samples were incubated at 32 °C for 1 h and centrifugated before being transferred to Standard Monolith NT™ Capillaries. The capillaries were scanned at 25 °C using the MST instrument (20% LED, medium MST power). For each compound, at least two independent experiments were performed. All data were analyzed using MO Affinity Analysis software (NanoTemper, Munich, Germany).

#### 3.2.12. Atomic Force Microscopy

The complexes obtained in the reaction between 8 µM peptidomimetics **5a**, **13a**, and **14a** and 2 nM p_max_GFP plasmid DNA (Lonza, Switzerland) were examined using AFM in 8 mM MgCl_2_ at room temperature in the PeakForce Tapping mode, using BioScope Resolve AFM (Bruker, Bremen, Germany). The ScanAsyst-Fluid+ probe (Bruker) was used for DNA-peptidomimetic complex imaging (resonant frequency f_0_ = 150 kHz; spring constant *k* = 0.7 N/m). Images were taken at 512 × 512 pixels with a PeakForce Tapping frequency of 1 kHz and an amplitude of 150 nm. Height sensor signal was used to display the protein image using NanoScope Analysis v1.9 (Bruker, Bremen, Germany).

## Figures and Tables

**Table 1:** Physicochemical characteristics of series **1** compounds along with their DNA binding constant, cell permeability, and cytotoxicity.

No	Sequence	Retention Time * [min]	Molecular Weight Calculated/Determined **	Cell Permeablility (Target) ***	Binding Constants Double [µM] ^#^	Cytotoxicity ^##^
					Single-Stranded DNA [µM] ^#^	
**1**	O2Oc(Arg)_6_-O2Oc-NH_2_	2.11	1244.5/1245.4	ND	Moderate	10%
**1a**	5′,6-FAM-O2Oc-(Arg)_6_-O2Oc-NH_2_	9.12/9.26	1603.8/1604.4	++ (cytoplasm)	7.4 ± 0.1	10%
					0.02 ± 0.04 ^&^	
**2**	O2Oc(D-arg)_6_-O2Oc-NH_2_	2.12	1244.5/1245.5	ND	Moderate	15%
**2a**	5′,6-FAM -O2Oc-(D-arg)_6_-O2Oc-NH_2_	8.46/8.71	1603.8/1604.8	++ (cytoplasm)	–	15%
**3**	O2Oc-(Har)_6_-O2Oc-NH_2_	2.45	1329.6/1330.4	ND	Weak	10%
**3a**	5′,6-FAM -O2Oc-(Har)_6_-O2Oc-NH_2_	8.55/8.83	1687.9/1689.0	++ (cytoplasm)	–	10%
**4**	O2Oc-[Dap(GO1)]_6_-O2Oc-NH_2_	3.15	1682.8/1683.6	ND	8.8 ± 2.1/	–
					1.8 ± 1.1 ^&&^	
**4a**	5′,6-FAM -O2Oc-[Dap(GO1)]_6_-O2Oc-NH_2_	7.89/8.13	2042.1/2043.0	++ (nucleus)	3.3 ± 2.6/	–
					1.7 ± 2.0 ^&^	
**4b**	5′,6-TAMRA-O2Oc-[Dap(GO1)]_6_-O2Oc-NH_2_	8.17/8.59	2096.2/2097.0	++ (nucleus)	ND	–
**5**	O2Oc-Dap(GO2)_6_-O2Oc-NH_2_	3.40	1947.1/1947.9	ND	1.8 ± 0.9/	–
					2.8 ± 1.2 ^&&^	
**5a**	5′,6-FAM-O2Oc-[Dap(GO2)]_6_-O2Oc-NH_2_	9.10/9.83	2306.4/2307.6	+++ (nucleus)	3.4 ± 3.0/	–
					2.3 ± 4.6 ^&^	
**5b**	5′,6-TAMRA-O2Oc-[Dap(GO2)]_6_-O2Oc-NH_2_	9.82/10.15	2360.6/2361.4	+++ (nucleus)	ND	–
**6**	O2Oc-[Dap(O2(GO1))]_6_-O2Oc-NH_2_	3.55	2554.7/2555.3	ND	ND	–
**6a**	5′,6-FAM-O2Oc-[Dap(O2(GO1))]_6_-O2Oc-NH_2_	12.99/13.43	2913.0/2913.8	–	ND	–
**7**	O2Oc-[Dap(O2(GO2))]_6_-O2Oc-NH_2_	4.09	2819.1/2819.9	ND	ND	–
**7a**	5′,6-FAM-O2Oc-[Dap(O2(GO2))]_6_-O2Oc-NH_2_	14.35/15.03	3177.3/3178.3	–	ND	–
**8**	O2Oc-[Dap(O2)]_6_-O2Oc-NH_2_	3.45	1694.9/1696.0	ND	Weak	–
**8a**	5′,6-FAM-O2Oc-[Dap(O2)]_6_-O2Oc-NH_2_	9.12/9.38	2053.2/2054.1	–	ND	–
**9**	O2Oc-[Dap(HO2)]_6_-O2Oc-NH_2_	3.71	1700.8/1701.5	ND	Weak	–
**9a**	5′,6-FAM-O2Oc-[Dap(HO2)]_6_-O2Oc-NH_2_	9.45/9.78	2059.1/2060.4	–	ND	–
**10**	5′,6-FAM	11.21	376.3	–	639 ± 703/	–
					399 ± 497 ^&^	
**11**	5′,6-TAMRA	12.17	431.5	–	ND	–

* Ultra performance liquid chromatography UPLC analysis (Nexera X2 LC-30AD (Shimadzu, Japan)) equipped with a Phenomenex column (150 × 2.1 mm), with a grain size of 1.7 µm (peptide XB-C18) equipped with a UV-Vis detector and a fluorescence detector. A linear gradient from **2** to 80% B within 15 min was applied (A: 0.1% trifluoroacetic acid; B: 80% acetonitrile in A); for fluorescent-labeled compounds, two retention times correspond to two diasereoisomers being provided; ** HR MALDI analysis with 2,5-dihydroxybenzoic acid as a matrix; *** confocal fluorescence microscope Olympus I51 (Olympus, Japan); ^#^ binding constant determined with single- or double-stranded DNA using microscale thermophoresis (MST) (labeled compounds) or surface plasmon resonance (SPR) (unlabeled compounds); ^##^ MTT assay for MDA-MB-231 or HB-2 cell lines (numbers indicate the percentage of dead cells) performed at the greatest concentration of 50 µg/mL; ^&^ MST binding constants; ^&&^ SPR binding constants; ND: not determined; “-“ means no cytotoxic effect was observed; “moderate” or “weak” DNA binding was aribitrary set based on the SPR experiment (Figure S3); weak: compounds below 10 RU, moderate: up to 60 RFU.

**Table 2:** Physicochemical characteristics of series **2** compounds, including their DNA binding constant, cell permeability, and cytotoxicity.

No	Sequence	Retention Time * [min]	Molecular Weight Calculated/Determined **	Cell Permeablility (Target) ***	Binding Constant Double DNA [µM] ^#^	Cytotoxicity ^##^
					Single-Stranded DNA [µM] ^#^	
**12**	O2Oc-[Dap(GO2)]_2_-O2Oc-NH_2_	1.94	853.9/854.6	ND	weak	–
**12a**	5′,6-FAM-O2Oc-[Dap(GO2)]_2_-O2Oc-NH_2_	10.11/10.43	1213.2/1213.5	–	–	–
**13**	O2Oc-[Dap(GO2)]_4_-O2Oc-NH_2_	2.09	1400.5/1401.2	ND	–	–
**13a**	5′,6-FAM-O2Oc-[Dap(GO2)]_4_-O2Oc-NH_2_	9.78/10.01	1759.8/1759.8	++ (cytoplasm)	0.3 ± 0.1/	–
					0.5 ± 0.2 ^&^	
**5**	O2Oc-[Dap(GO2)]_6_-O2Oc-NH_2_	3.40	1948.1/1947.1	ND	1.8 ± 0.9/	–
					2.8 ± 1.2 ^&&^	
**5a**	5′,6-FAM-O2Oc-[Dap(GO2)]_6_-O2Oc-NH_2_	9.10/9.83	2306.4/2307.6	+++ (nucleus)	3.4 ± 3.0/	–
					2.3 ± 4.6 ^&^	
**5b**	5,6-TAMRA-O2Oc-[Dap(GO2)]_6_-O2Oc-NH_2_	9.82/10.15	2360.6/2361.4	+++ (nucleus)	ND	–
**14**	O2Oc-[Dap(GO2)]_8_-O2Oc-NH_2_	2.97	2493.7/2493.5	ND	ND	10%
**14a**	5′,6-FAM-O2Oc-[Dap(GO2)]_8_-O2Oc-NH_2_	8.2/8.8	2852.0/2853.7	+++ (nucleus)	0.2 ± 0.1/	15%
					3.3 ± 2.34 ^&^	
**14b**	5′,6-TAMRA-O2Oc-[Dap(GO2)]_8_-O2Oc-NH_2_	8.2/8.5	2907.1/2908.5	+++ (nucleus)	ND	15%

* UPLC analysis (Nexera X2 LC-30AD (Schimadzu, Japan)) equipped with a Phenomenex column (150 × 2.1 mm), with a grain size of 1.7 µm (peptide XB-C18) equipped with a UV-Vis detector and a fluorescence detector. A linear gradient from 2 to 80% B within 15 min was applied (A: 0.1% trifluoroacetic acid; B: 80% acetonitrile in A); for fluorescent-labeled compounds, two retention times correspond to two diasereoisomers being provided; ** HR MALDI analysis with 2,5-dihydroxybenzoic acid as a matrix; *** confocal fluorescent microscope Olympus IX51 fluorescence microscope (Olympus, Japan); ^#^ binding constant determined with single strand ss or double strand dsDNA using MST (labeled compounds) or SPR (unlabeled compounds); ^##^ MTT assay for MDA-MB-231 or HB-2 cell lines; ^&^ MST binding constants; ^&&^ SPR binding constants; ND: not determined; “-“ means no cytotoxic effect was observed; “weak” DNA binding was aribitrarily set based on the SPR experiment (Figure S3).

## Data Availability

Not applicable.

## Supplementary Materials

The following are available online at https://www.mdpi.com/1422-0067/22/5/2571/s1.
