# Supplementary material for: Novel Cell Permeable Polymers of N-Substituted L-2,3-Diaminopropionic Acid (DAPEGs) and Cellular Consequences of Their Interactions with Nucleic Acids
Source: Int J Mol Sci. 2021 Mar 4;22(5):2571. doi: 10.3390/ijms22052571 (PMC7961587; doi:10.3390/ijms22052571)
Supplement: Supplementary file 1 [file ijms-22-02571-s001.pdf]

Supplementary Materials

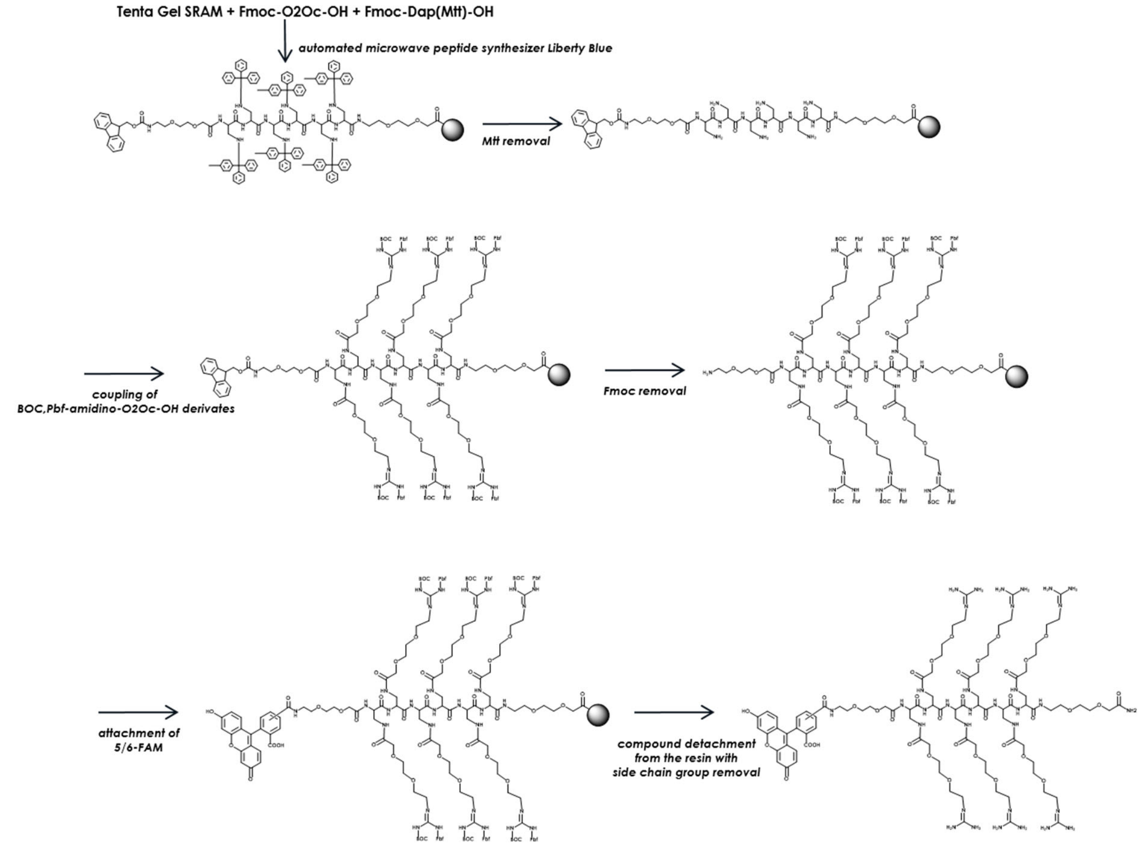

Figure 1S. Scheme of synthesis of compound 5a

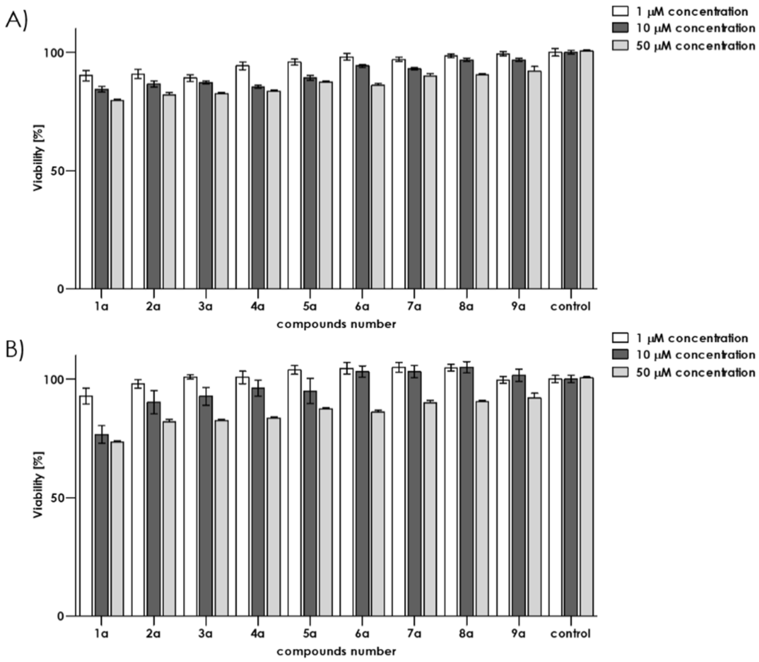

Figure 2S. Cytotoxicity of compounds 1a–9a as assessed by the MTT assay. Two different cell lines were used: A) HB-2 and B) MDA-MB-231.

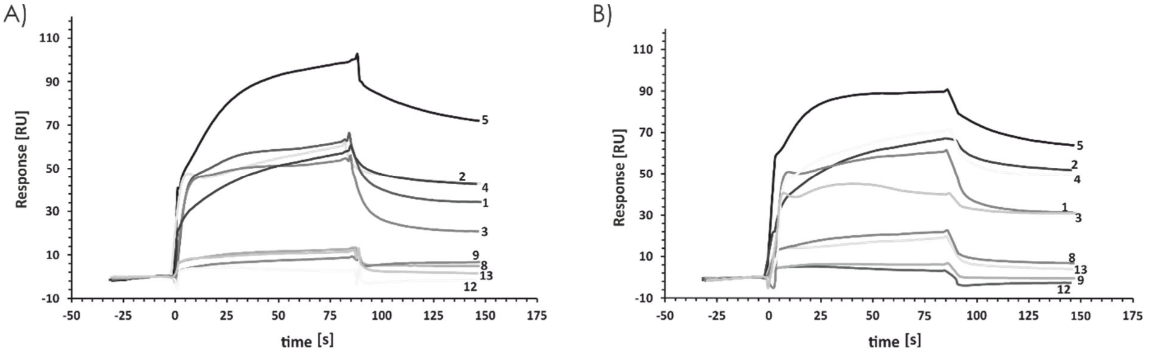

Figure 3S. Surface plasmon resonance SPR analysis of double-stranded DNA (dsDNA) binding by various peptidomimetics. The SPR analysis of dsDNA binding was performed using: A) 50 μM and B) 100 μM of the indicated compounds, flowed over the SA sensor chip surface with immobilized 76 bp biotinylated DNA fragment containing the sequence of beta-actin (*Homo sapiens*). Injections were performed in HBS-EP buffer. HBS-EP was also used as the running buffer.

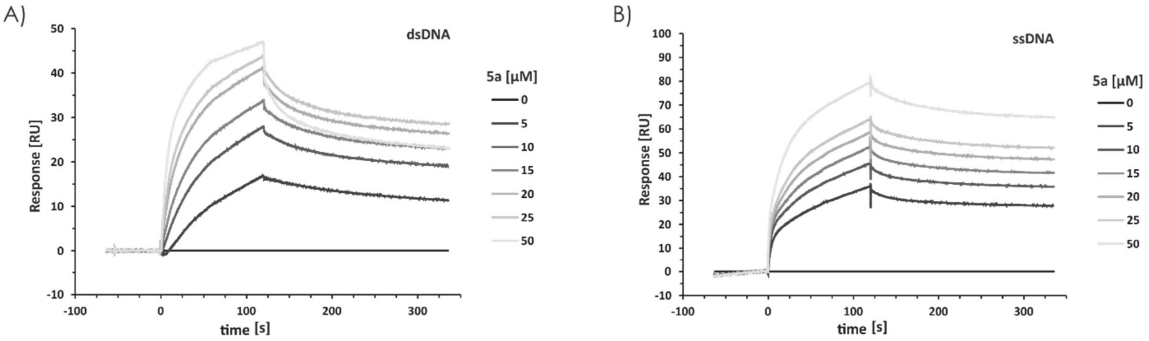

C)

	ka	SD	kd	SD	KD	SD
dsDNA	4.58e02	1.77e02	8.01e-04	2.38e-04	1.81e-06	2.63e-07
ssDNA	6.23e02	2.81e02	1.69e-03	1.42e-03	2.78e-06	2.14e-06

Figure 4S. SPR analysis of DNA binding by compound 5a. The SPR analysis of double-stranded DNA (dsDNA; A) and single-stranded DNA (ssDNA; B) binding by compound 5a was performed using SA sensor chip surface with immobilized 76 bp or 76 nt biotinylated DNA fragment containing the sequence of beta-actin (*Homo sapiens*). An increasing concentration of compound 5a was used, as indicated above. Injections were performed in HBS-EP buffer. HBS-EP was also used as the running buffer. (C) Kinetic constants calculated from at least three experiments using Biacore T200 Evaluation Software

Table 1S. Minimal charge to ratio (N/P) of each compound for efficient complexation with DNA. *minimal N/P ratio resulting in effective complex formation based on EMSA. **ND - no determinated

No	Sequence	Minimal N/P ratio*
**1**	O2Oc(Arg)$_6$-O2Oc-NH$_2$	1.5:1
**2**	O2Oc(D-arg)$_6$-O2Oc-NH$_2$	1.5:1
**3**	O2Oc-(Har)$_6$-O2Oc-NH$_2$	1.5:1
**4**	O2Oc-[Dap(GO1)]$_6$-O2Oc-NH$_2$	0.2:1
**5**	O2Oc-Dap(GO2)$_6$-O2Oc-NH$_2$	0.2:1
**6**	O2Oc-[Dap(O2(GO1))]$_6$-O2Oc-NH$_2$	1.5:1
**7**	O2Oc-[Dap(O2(GO2))]$_6$-O2Oc-NH$_2$	1.5:1
**8**	O2Oc-[Dap(O2)]$_6$-O2Oc-NH$_2$	1.5:1
**9**	O2Oc-[Dap(HO2)]$_6$-O2Oc-NH$_2$	No binding
**12**	O2Oc-[Dap(GO2)]$_2$-O2Oc-NH$_2$	ND**
**13**	O2Oc-[Dap(GO2)]$_4$-O2Oc-NH$_2$	1.5:1
**14**	O2Oc-[Dap(GO2)]$_8$-O2Oc-NH$_2$	0.2:1

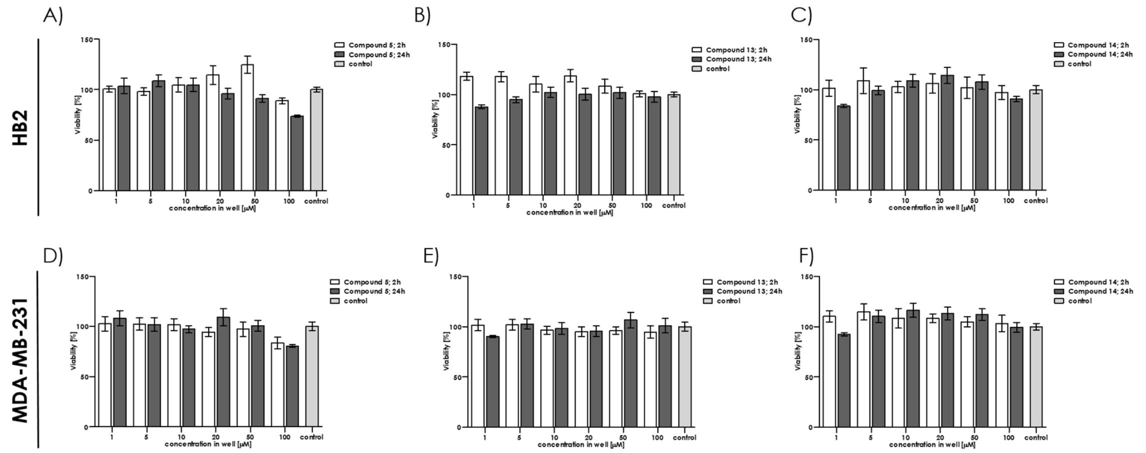

Figure 5S. CCK8 cytotoxicity assay performed for three compounds (5, 13, and 14). Two different cell lines were used: A) HB-2 and B) MDA-MB-231
